# Human reaction time in a mixed reality environment

**DOI:** 10.3389/fnins.2022.897240

**Published:** 2022-08-19

**Authors:** Syed Muhammad Umair Arif, Michele Brizzi, Marco Carli, Federica Battisti

**Affiliations:** ^1^Department of Industrial, Electronic and Mechanical Engineering, Roma Tre University, Rome, Italy; ^2^Department of Information Engineering, University of Padova, Padova, Italy

**Keywords:** mixed reality, human computer interaction, reaction time, saliency, quality of experience

## Abstract

Over the last few years applications based on the use of immersive environments, where physical and digital objects coexist and interact, have gained widespread attention. Thanks to the development of new visualization devices, even at low cost, and increasingly effective rendering and processing techniques, these applications are reaching a growing number of users. While the adoption of digital information makes it possible to provide immersive experiences in a number of different applications, there are still many unexplored aspects. In this work, a preliminary step to understand the impact of the scene content on human perception of the virtual 3D elements in a mixed reality has been performed. To this aim, a subjective test was designed and implemented to collect the reaction time of a set of users in a mixed reality application. In this test each user was asked to wear an augmented reality headset and to catch a virtual objects randomly appearing in the subject's field of view. We first estimated the detection accuracy through omitted, anticipated, and completed responses; then we related stimulus location, scene content and estimated accuracy. For this purpose, the area of stimulus presentation was divided into upper, lower, right, left, inner, and outer, to understand in which area responses were omitted and anticipated with respect to the central point of view. Experimental results show that, in addition to the saliency of the real scene, natural body gesture technology and limited field of view influenced human reaction time.

## 1. Introduction

In the late 90s, Paul Milgram and Fumio Kishino defined the *virtuality continuum*, a continuous scale encompassing completely real and completely virtual environments, to categorize visual interfaces capable of merging them (Milgram and Kishino, 1994). Based on this taxonomy, Mixed Reality (MR) allows to create a unique three-dimensional real-time environment that enhances user perception through extended interaction capabilities with virtual objects inside a real environment. Immersive technologies provide a new direction for users and researchers to explore either real or virtual 360° environments with a Head Mounted Display (HMD). For these reasons, MR, Augmented Reality (AR), and Virtual Reality (VR) have been employed in a number of applications from the medical field (Barrie et al., 2019; Gao et al., 2019; Hu et al., 2019; Gerup et al., 2020; Halder, 2022), virtual training (Brunnström et al., 2018; Kong et al., 2018; Hynes et al., 2019), virtual tourism (Baran and Baran, 2022), art and design (Du, 2021), construction and design process (Calderon-Hernandez et al., 2019; Orihuela et al., 2019; Tabrizi and Sanguinetti, 2019), and environmental simulations (Sermet and Demir, 2022).

In this paper we investigate the possibility of adopting MR technologies in emergency management, such as safety-critical control room operations. In this scenario, the presence of humans in the decision-making cycle is required. Anyway, additional information may be given to the operator by mixing computer generated data with the real environment in order to improve operator performance. Physically realistic objects, registered in space so that they are perceived as within reach in the real environment, can be presented to the user through a virtual control interface and used to supplement the visual information that would otherwise be supplied by means of traditional 2D interfaces.

Supervising or managing a control room is a demanding task, that requires both good accuracy and high speed of execution, corresponding to a considerable mental load, which could be reduced with the help of the virtual elements. The types of interactions and the quality of the scene contents are very different from what can be experienced by visualizing the same information through conventional displays, even if compared with other interactive manners like using VR HMDs. The MR environment requires the cooperation of multiple cognitive functions, i.e., attention, visual perception, memory, learning, and motor skills to retrieve relevant information. Moreover, since a mixed reality application includes both real and artificial environments, it is necessary that the user maintains attention to elements from both worlds at the same time. Consequently, there is a risk of overloading the user thus delaying the decision-making process. Moreover, the real-world background could have an impact on the visibility of the virtual-world foreground element, hampering the promptness of the response. In this regard, the study of the total reaction time, defined as the time needed for perceiving the visual stimuli, processing the information, making a decision, and providing an appropriate response, may be useful for understanding the usability of MR technologies for these purposes. In this context, we focus on the analysis of the human reaction time in a MR environment to verify the suitability of these techniques in environments that require a quick response. In general, users are more familiar with traditional human-computer interaction modalities (i.e., keyboard and mouse) thus resulting in a shorter response time. Thus, in order to evaluate MR interfaces, a few considerations should be made.

First, a typical MR HMD usually allows several ways to interact with the mixed reality environment, such as hand gestures (also known as mid-air gestures), gazes, or voice commands. Each interaction mode is characterized by different reaction times, that account for eye motor skills, eye coordination timing, neck motor response time, and arm motor response time, plus the time needed to identify the target and the cognitive processing time to provide the appropriate response. Moreover, compared to the Human Visual System (HVS), available MR devices have narrower Field of View (FoV). For this reason, especially when auditory and visual cues are not present to direct the attention, users require more time to visualize spatially-displaced information in the mixed reality environment as compared to 2D interfaces.

In this work, we evaluate the reaction time of subjects wearing MR headsets. To this aim, a MR interface for rendering the visual stimuli was designed. While traditional human attention tests, using 2D display, keyboard and mouse interaction systems, have been shown to be a reliable way for measuring human reaction times for cognitive tasks during clinical trials, to the best of our knowledge only a few studies have addressed the problem of measuring human performance parameters such as accuracy and visual processing speed of users in a MR environment. The detection accuracy was estimated through omitted, anticipated, and completed responses. Moreover, we related stimulus location, scene content and estimated accuracy. For this purpose, the area of stimulus presentation was divided into upper, lower, right, left, inner, and outer, to understand in which area responses were omitted and anticipated with respect to the central point of view.

This contribution will focus on the test methodology for recording the reaction times considering an air-tap interaction modality (Microsoft, 2019a), the dataset of reaction times based on the MR task, and a statistical analysis carried out to understand the factors that may affect the reaction time of a user in a MR application, will be addressed in this contribution. Moreover, the dependency on the scene content, especially on the background where virtual objects are placed, is evaluated by considering the visual saliency of the real world scenes, with and without the presence of virtual elements.

The remainder of the paper is organized as follows: in Section 2 related works on reaction time tests, for both the traditional 2D case and the MR case, are discussed. Section 3 describes the methodology used to collect the MR reaction time. In Section 5, the outcomes of the experiment are presented. Finally, in Section 6, the conclusions are drawn.

## 2. Related work

High-level cognitive functions (i.e., attention, alertness, and visual perception) are associated with the time needed to process visual stimuli and to transmit the processed information to the brain (Nobre et al., 2014). For this reason, the study of the reaction time has been widely addressed in literature.

### 2.1. Reaction time for 2D interfaces

Zimmermann studied intensity aspects of attention such as alertness and vigilance by exploiting human reaction time (Zimmermann and Fimm, 2002). Alertness was measured by providing visual stimuli to the subject, who had to respond in a given time. To measure the intensity aspects of attention, several computer-based tests have been developed. Posner and Boies (1971) exploited the attention aspects with experiments and subdivided them into distinguishable components, i.e., alertness, selectivity, and processing capacity. Pachella (1973) exploited information processing study with human reaction time. Reaction time is also dependent on the logic and design of experimental study and hardware interaction modalities. Interpretation of reaction time depends on various steps, i.e., time of recognition, time to deduce, decision time, and response time. Botwinick and Thompson (1966) interpreted reaction time with motor components of reaction time, i.e., pre-motor time, motor time, and total reaction time evaluated by employing EEG and EMG devices during the experiment.

Hershenson (1962) performed a subjective study to compare the human reaction time when using three types of stimuli: light only, sound only, and light with sound. In this work, it was observed that low-intensity light was difficult to perceive and, as a result, the human response was slower than in the task in which only sound was used or in presence of light with higher intensity. Deary et al. (2011) developed an open-source software to measure the reaction time in two commonly used types of task: Simple Reaction Time (SRT) in which the subject is asked to respond as quickly as possible to a stimulus, and Choice Reaction Time (CRT) for which there are two or more possible stimuli requiring different responses. Chen et al. (2021) developed two *serious games* to study the differences in reaction time and accuracy. Each game was designed with increasing difficulty levels. Bøttern et al. (2009) investigated visual and auditory reaction time in presence of incongruent and congruent distractors. The Flanker task was adopted and modified with low, medium, and high workloads. It was shown that the reaction time is higher in more complex tasks compared to low complexity ones. Wells et al. (2014) developed a device for the assessment of reaction time, the visuomotor. In the state-of-the-art, the reaction time has been used for applications in several fields. Chen et al. (2017) performed a comparative subjective study to analyze the performance of cognitive functions by measuring the reaction time of three groups of subjects: affected by Mild Cognitive Impairment (MCI), affected by Alzheimer's disease, and healthy controls. In previous studies, multiple reaction time experiments were performed to study human behavior, i.e., simple, recognition, choice, and serial to evaluate different human performance parameters. In SRT experiments, only one (either acoustic or spatial) stimuli and one response is allowed. In recognition experiments, multiple stimuli appear, with distractors among them, and subject must respond to the correct one. In CRT experiments, specialized hardware or different buttons are required to provide a response, but there is always choice to give a response with any provided button. In the serial reaction time, stimuli are presented in a fixed order instead of randomly (Kosinski, 2008). Multiple researchers proposed various methods to analyze attention intensity aspects by measuring human reaction time. Earlier, Galton (1890) performed a extensive subjective study to analyze the difference of sound and light stimuli on reaction time. The observation revealed that light stimuli reaction time is higher than acoustic stimuli. Moreover, Solanki et al. (2012) also concluded that visual stimuli have higher reaction time as compared to acoustic stimuli. Color of the stimuli is also correlated with the human reaction time. Balakrishnan et al. (2014) observed that the reaction time of red and green stimuli is less than yellow color stimuli. All executive control functions such as intensity aspects of attention, alertness, reaction time, cognitive set of abilities helps in a daily life for information processing and in a decision making process (Zhang et al., 2021). Few of the clinical psychological test studies are adopted to solve real world problems. For example, Moessinger et al. (2021) proposed a multi-modal based method, which included EEG and TAP attention performance test (Zimmermann and Fimm, 2002) to measure the driver's alertness in the night time and day time driving. Main goal of the study is to continuous monitoring of subject alertness level during long hours of drivings in the nighttime that affects human performance leads to dangerous situations and cause many accidents.

### 2.2. Reaction time for MR interfaces

Traditional human attention test methods are more reliable for measuring human reaction time in clinical tasks with traditional display rendering devices and keyboard and mouse interaction modalities or interaction modalities designed for a specialized use. To the best of our knowledge, only few studies addressed the problem of measuring human performances in a mixed reality environment in terms of accuracy and processing speed.

Webb et al. (2016) performed a subjective study to analyze user performance, accuracy, and psycho-physiological with multiple AR devices. Multiple devices and multiple test protocols were designed to record human responses. Investigation revealed that response time was much longer for AR devices as compared to traditional devices. Batmaz et al. (2020) investigated human eye-hand reaction time with AR, VR, and 2D touch screens. The reaction time VR and 2D touch screens was found to be shorter than AR reaction time due to gestures. Antão et al. (2020) developed an AR-based game and performed a comparative study of Autism Spectrum Disorder(ASD) and healthy control subjects by analyzing the differences in reaction time while performing specific tasks. Vlahovic et al. (2021) performed a subjective study to analyze the discomfort, cybersickness, and reaction time on the use of virtual reality. To this aim, two reaction time tests were used: the Deary-Liewald Reaction Test (DLRT) to measure the SRT, and a four-CRT. Arif et al. (2021) performed a subjective study on motor skills processing speed in a mixed reality environment by measuring the reaction time. The authors developed a mixed reality application to measure the SRT. Human performance (i.e., mean and standard deviation of reaction time) and accuracy (i.e., omitted, completed, and anticipated responses) metrics were used to analyze the reaction time. Kourtesis et al. (2021) developed a virtual reality-based game “VR-EAL” to simulate real-life situations for the assessment of cognitive functions such as prospective memory, episodic memory, and attention. Finally, Mifsud et al. (2022) assessed the impact of hologram transparency on selection in AR.

## 3. Experimental setup

As mentioned in the Introduction, in this work we aim at understanding the impact of the virtual objects and of the real environment in a MR application based on the analysis of the reaction time. To this aim, we prepared an experimental environment and developed a SRT mixed reality application to collect the reaction time, as detailed in the following.

### 3.1. Laboratory environment preparation

**Figure 1:**
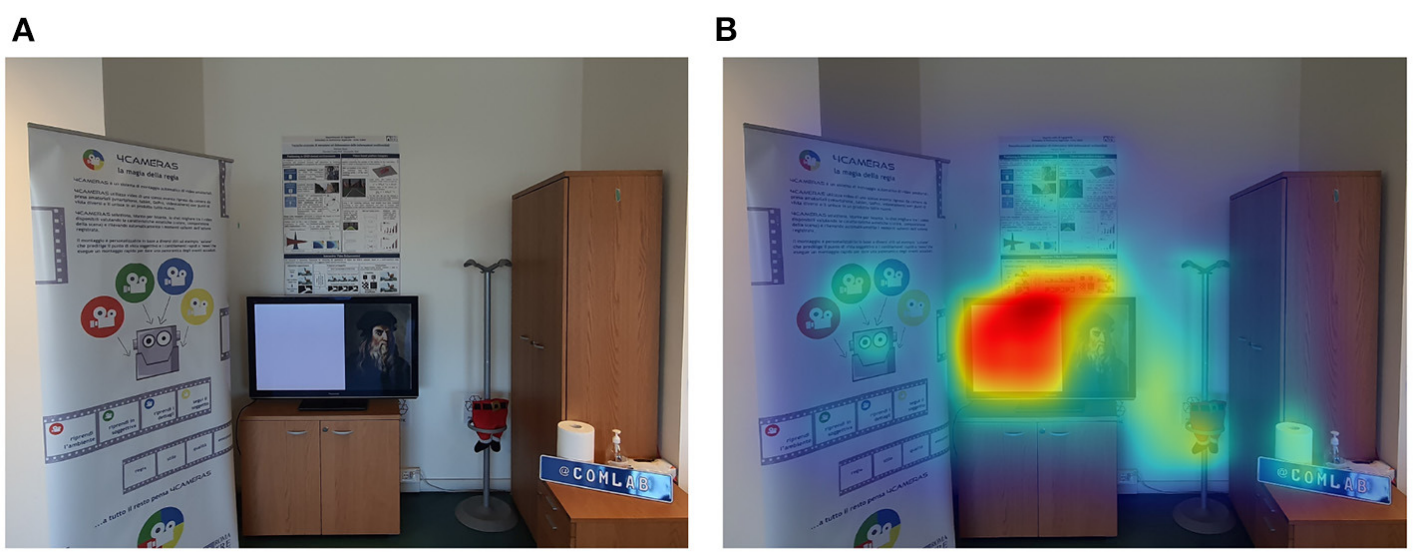
**(A)** Laboratory environment and **(B)** output of Graph-Based Visual Saliency method (Harel et al., 2006).

The first step of our proposed work is to set up a cluttered view scene in the immediate FoV of the subject. We inserted several objects to simulate a real world scenario in which a MR application is used without any requirement or restriction. To this aim, a starting position has been defined in the real environment so that all subjects could see the same scene. Then, wall and stand posters, a 32 inches UHD TV with a digital photo, cabinets and other real objects were placed in front of the starting position as shown in Figure 1A. The background and the immediate surroundings of the test environment were not changed throughout the performance of the experiments. The experiment was conducted in a controlled light environment in order to ensure the visibility of the holograms displayed through the MR HMD, whose display is rated for ~350*cd*/*m*^2^.

In order to estimate the impact of the clutter on the reaction time, we computed the saliency of the scene (that is, which areas of the image are more likely to attract the attention of the user) using the method from Harel et al. (2006). The result, shown in Figure 1B, indicates the salient regions of the cluttered scene.

### 3.2. Mixed reality application and architecture

**Figure 2:**
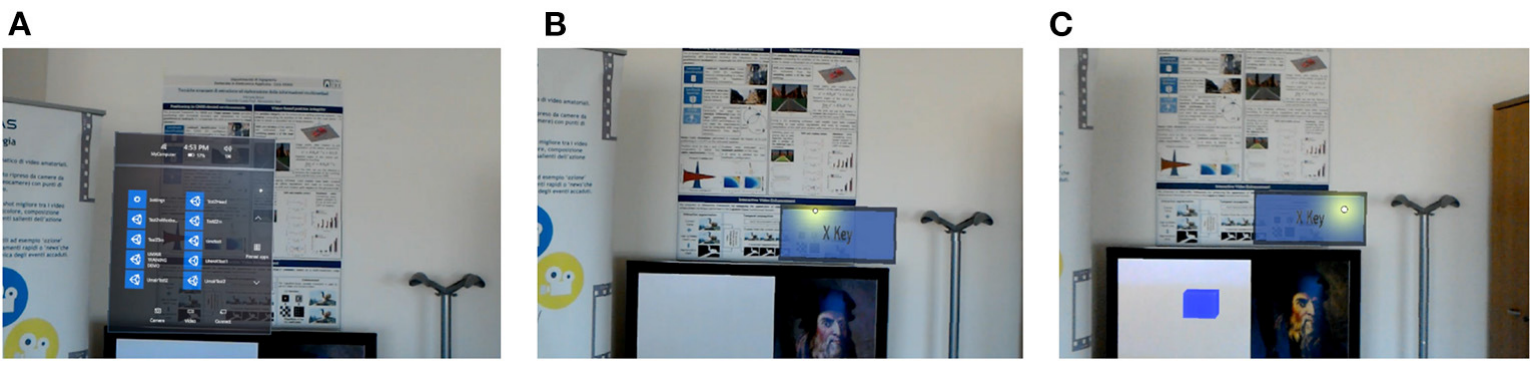
Mixed Reality Simple Reaction Time User Interface, showing, **(A)** the device menu prior to starting the test, **(B)** the X-KEY button, and **(C)** one of the visual stimuli and the X-KEY button being selected by the user.

The second step is to develop a SRT application for recording human reaction time in a mixed reality environment. To this aim, we developed the SRT application rendered by using Microsoft HoloLens (Kress and Cummings, 2017) HMD. The Mixed-Reality-Tool-kit (MRTK) package (Microsoft, 2019b) has been used to design the 3D User Interface (UI) in Unity 3D (Unity3D, 2019). Microsoft world-lock recommendations have been used, to lock user interface on one spatial location as compared to user head movements inside the mixed reality environment (Microsoft, 2019c). The UI design consists of visual stimuli in the form of colored cube-shaped holograms, and a button (X-KEY) that should be pressed by the user whenever a stimulus appears. An example of the Mixed Reality SRT UI, as seen from the user point of view, is shown in Figure 2.

**Figure 3:**
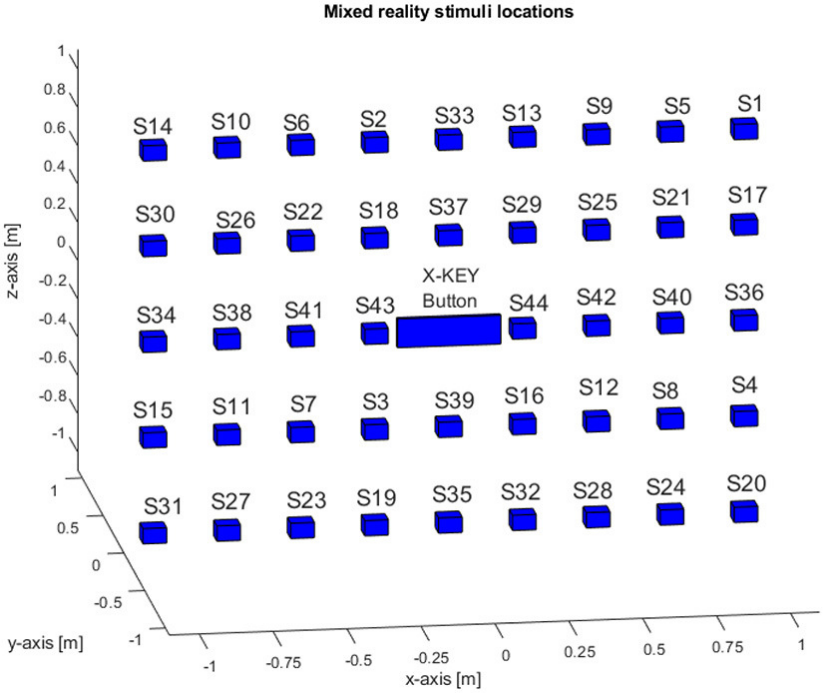
Stimuli numbers and spatial locations chart in a mixed reality environment.

In the SRT task, a total of 44 stimuli were presented. They were set to randomly appear at uniformly-spaced spatial coordinates, spanning an area of 2 × 2 m in the mixed reality environment. More specifically, the stimuli locations were arranged on a rectangular grid of 9 by 5. The stimuli on each row were disposed at an horizontal distance of 0.25 m from each other, and the vertical distance between rows was 0.5 m. The size of each stimulus was 8 × 6 × 7 cm. The spatial locations chart of the virtual stimuli is shown in Figure 3. The central position was occupied by the X-KEY button, which has a size of 30 × 12 × 3 cm. The X-KEY button and the stimuli were displayed at a distance of 2.2 m from the observers' starting position.

Subjects could freely move their head to locate the virtual stimuli within the mixed reality environment. For interacting with the MR interface and the X-KEY button, hand gestures (air taps) were selected (Microsoft, 2019a). Each time a virtual stimulus appeared, the participant had to respond by pressing the X-KEY button as quickly as possible. Each stimulus remained visible until the button was pressed, after which the following stimulus appeared in a different position. However, a time limit of 5 s was allocated for each stimulus. Depending on the promptness of the subject in responding to the stimulus, three possible outcomes are considered in the experiment: (i) correct: the stimulus was detected and the subject pressed the X-KEY button on time; (ii) anticipated: the subject pressed the X-KEY button before the stimulus was displayed; and (iii) omitted: the stimulus was not detected within 5 s or the user pressed the X-KEY button after the time limit.

**Figure 4:**
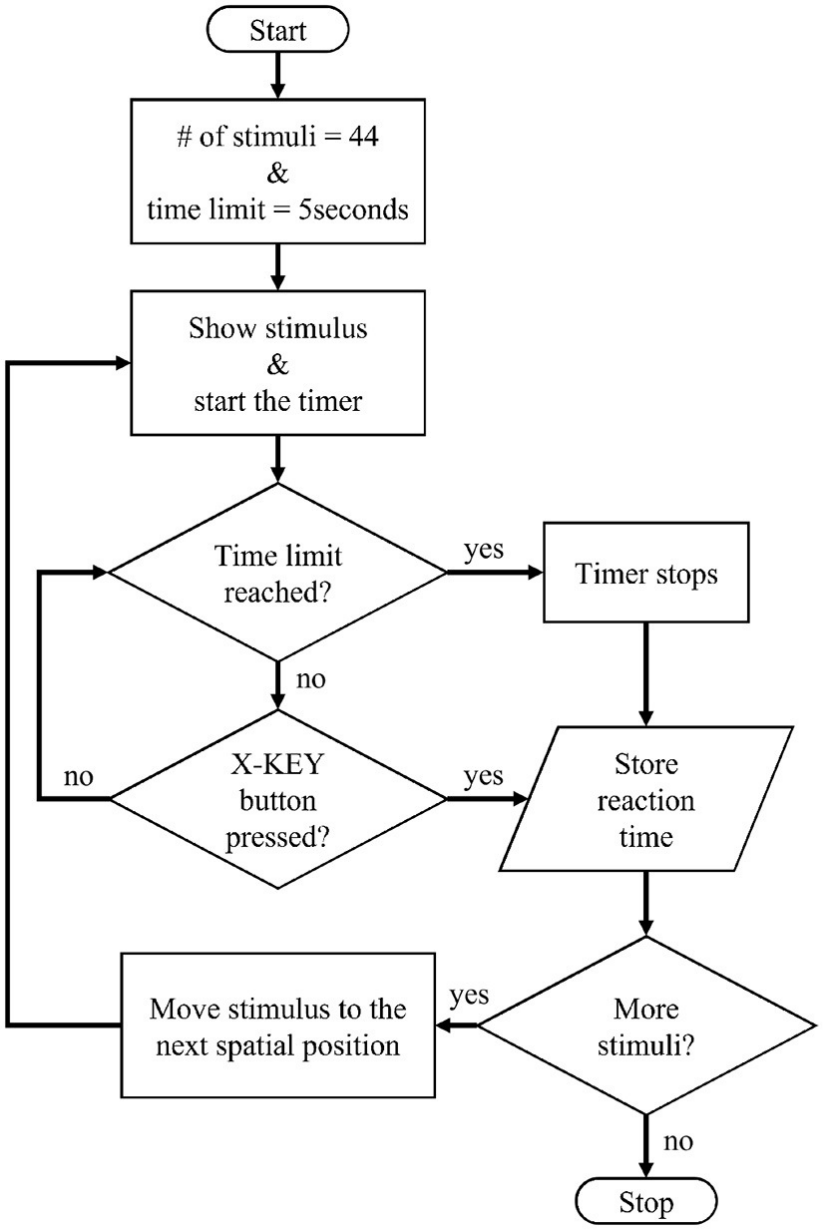
Flowchart of the mixed reality SRT application.

A flowchart of the mixed reality SRT application is shown in Figure 4. The application records the response time of the subject to each stimulus and whether the response was correct, anticipated, or omitted.

## 4. Subjective experiment

A total of 16 subjects participated to the SRT experiment. Subjects were recruited among students and personnel of Roma Tre University. The age range of the subjects is between 22 and 40 years (mean = 25.93, std = 5.43). The test was performed according to the Recommendation ITU-T P.800.1 (ITU, 2006). On average, the length of the test was 50 min. The protocol has been divided into three phases which will be detailed in the following.

### 4.1. Information and screening phase

Each subject signed a written consent form to participate in the experiment. Questionnaires were designed to collect demographic and information on previous experience with MR technologies from the subjects. All participants were tested for visual acuity using the Snellen chart (Snellen, 1863), and for color perception through the Ishihara test (National Research Council, 1981). The requirements to participate to the test were to pass the Snellen test with at least a 20/40 score, and not to be color blind.

### 4.2. Training phase

In the training phase, written and verbal instructions regarding the test were provided to the subject. The training phase is divided into two sub-steps. In the first, we provided a basic training on the use of the MR HMD to allow the viewer to get familiar with mixed reality environments and interactions. This phase lasts for 20 min. In the second part of the training phase, to get familiar with the SRT application interface, subjects were asked to follow the task instructions. This phase lasts for 10 min. Results collected during this phase were not considered in the evaluation. Additional 5 min of rest were given to the subjects at the end of the training phase before the beginning the real test.

### 4.3. Testing phase

In the testing phase, each subject was asked to stand at the pre-defined test start position, at a known distance from the MR contents, and to face toward the cluttered environment in front of which the MR contents were displayed. They were instructed to open the HMD device menu, to start the SRT test application, to find the stimuli, and to provide the appropriate response on the X-KEY button as quickly as possible. The first stimulus was set to appear 20 s after the start of the testing phase, and all the following stimuli were set to appear after a 5 s interval. The testing phase lasted for a total of 5 min.

## 5. Results and discussion

Following the Recommendation ITU-T P.800 (ITU, 2006), an outliers detection procedure was applied to the collected reaction time data set, and no outliers were found. In the testing phase, a total of 44 stimuli were presented. However, the data collected for the last 4 stimuli were not included in the results to avoid the impact of fatigue on the reaction times. Then, we computed the mean and standard deviation of the reaction times. For each subject, the mean value of the reaction time indicates the time that was required, on average, to respond to the stimuli during the task. Reaction time standard deviation reflects the variations in the attained speed to complete the task. To analyze subjects' individual performance, together with the reaction time we also computed the accuracy of the subjects in performing the task. To this aim, three possible response types, i.e., *completed, omitted*, and *anticipated*, were selected as defined in Arif et al. (2021). The results for the accuracy and reaction time are presented in Table 1.

**Table 1:** Reaction time and accuracy for each subject.

**Subject**	**Parameters of accuracy**			**Parameter of speed [reaction time (s)]**	
	**Anticipated**	**Omitted**	**Completed**	**Mean**	**Standard deviation**
Subject 1	2	4	34	3.00	1.16
Subject 2	1	5	34	3.07	1.35
Subject 3	4	8	28	3.01	1.45
Subject 4	1	1	38	2.56	1.02
Subject 5	2	1	37	2.09	0.93
Subject 6	1	2	37	2.51	1.17
Subject 7	6	16	18	3.66	1.68
Subject 8	3	9	28	3.45	1.36
Subject 9	6	8	26	2.95	1.58
Subject 10	6	13	21	3.36	1.70
Subject 11	5	4	31	2.69	1.35
Subject 12	0	2	38	2.65	1.17
Subject 13	3	8	29	2.99	1.51
Subject 14	3	5	32	2.50	1.33
Subject 15	0	0	40	2.50	0.84
Subject 16	2	4	34	3.32	1.39

Let us first consider the results regarding the accuracy. As clearly shown in Table 1, there is some variability in the responses given by the subjects, that can be attributed to the different visual and motor skills in issuing a reaction through the MR interface. Only one subject (subject no. 15) did not show anticipated or omitted responses, and succeeded in responding to all stimuli, showing great accuracy in completing the task. The same subject also achieved the best average response time, with the smallest mean value and standard deviation value. Similarly, subject no. 4 (who scored one omitted and one anticipated response) and subject no. 12 (who showed two omitted but zero anticipated responses) have in total 38 correct responses out of 40. Moreover, as we can see subject no. 7 and subject no. 10 have the most omitted responses as compared to the rest of the subjects, which reflects the poor accuracy in completing the task.

Let us now consider the reaction time of the subjects. As we can see reaction time of subjects is generally longer as compared to the 2D scenario (Zimmermann and Fimm, 2002; Deary et al., 2011). As can be noticed from Table 1, subject no. 5 responded with the shortest average time of 2.09 s and standard deviation of 0.93 s, values that reflect the subject's speed in completing the task. Similarly, subject no. 7 responded with average value 3.66 s and variation in the responses of 1.68 s.

To test whether the accuracy of the subjects and the reaction time were related, we computed the Pearson's linear correlation coefficient between the average reaction time and its standard deviation, and the number of *anticipated, omitted* and *completed* stimuli. The results are shown in Table 2. As expected, there is a strong relation between reaction time and accuracy.

**Table 2:** Pearson's linear correlation coefficient and *p*-values for hypothesis testing with regard to the relation between reaction time and accuracy.

**Test**	**ρ**	***p*-value**
React. time vs. anticipated	0.52	0.0386
Std react. time vs. anticipated	0.82	0.0001
React. time vs. omitted	0.82	9.841e-05
Std react. time vs. omitted	0.90	2.463e-06
React. time vs. completed	−0.76	0.0007
Std react. time vs. completed	−0.91	1.04e-06

More in detail, we found a high negative correlation of −0.76 and −0.91, with *p*-values much smaller than the significance value of 0.05, indicating rejection of the hypothesis that no correlation exists between the number of *completed* stimuli and the reaction time and its standard deviation, respectively. In addition, we also found a high positive correlation of 0.82 and 0.90, with *p*-values much smaller than the significance value of 0.05, indicating rejection of the hypothesis that no correlation exists between the number of *omitted* stimuli and the reaction time and its standard deviation, respectively. Finally, while a high positive correlation of 0.82 exists between the number of *anticipated* stimuli and the standard deviation of the reaction time, a smaller yet still significant correlation is found for the reaction time itself (0.52).

We argue that the saliency of the real environment is one of the important factors influencing human performance in mixed reality environments where physical and virtual objects co-exist. In fact, physical objects position, orientation, colors, contrast, graphical information, and textual information might attract the subjects' attention during the mixed reality operation, diverting it from the task. To this aim, we setup the laboratory environment with graphical information, textual information, placed the posters on different positions and orientation and other objects as shown in Figure 1. In the following, each stimulus is identified with a number as shown in Figure 3.

We further subdivided the space where the stimuli were set to appear into six sub-areas: (i) upper stimuli area (containing the topmost row of stimuli, i.e., S1, S5, S9, S33, S2, S6, S10, S14), (ii) lower stimuli area (containing the lowest row of stimuli i.e. S31, S27, S23, S19, S35, S32, S28, S24, S20), (iii) right stimuli area (containing the remaining stimuli of the rightmost column of stimuli, i.e., stimuli S17, S36, S4), (iv) left stimuli area (containing the remaining stimuli in the leftmost column of stimuli, i.e., stimuli S30, S34, S15), (v) inner upper stimuli area (containing stimuli S26, S22, S18, S37, S29, S25, S21), and (vi) inner lower stimuli area (containing stimuli S38, S39, S41, S43, S44, S42, S40).

**Figure 5:**
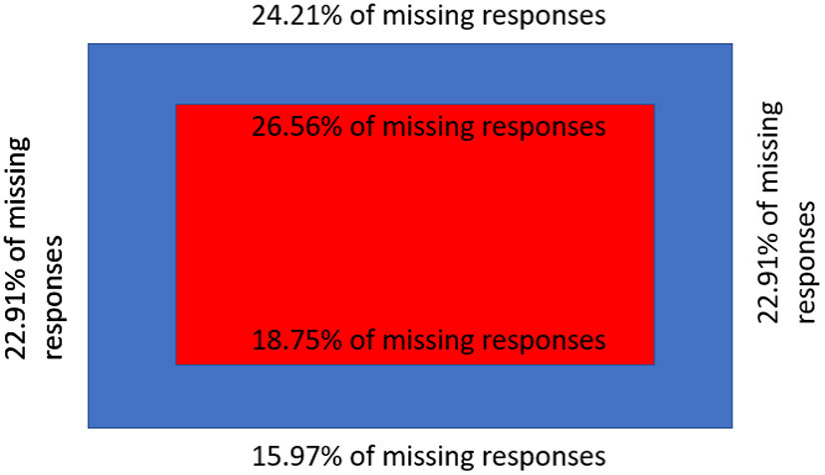
Percentage of missing responses in physically defined area.

In this regard, we analyzed the percentage of the missed stimuli responses of subjects (i.e., stimuli which were anticipated or omitted) in different sub-areas. Results of the missed responses analysis are shown in Figure 5. As we can observe, due to the saliency of the real scene (which is mainly focused on the graphical poster and UHD TV), the number of subjects' missed responses from the upper stimuli sub-area and the inner upper sub-area is higher than the number of missed responses from the lower stimuli and inner lower stimuli sub-areas. Right and left sub-areas, on the other hand, have the same percentage of missed responses. The higher number of missed responses for sub-regions (i), (ii), (iii), and (iv) could also be explained by the longer time which is required to perform the head movements needed for identifying visual stimuli placed outside of the FoV, which is another factor affecting the reaction time and the accuracy of the interaction.

Furthermore, we analyzed the average responses, over all subjects, of the individual stimuli for all anticipated, omitted and corrected values. We observed that no one of the subjects anticipated the stimuli S1, S8, S9, S12, S14, S18, S24, S26, S27, S28, S31, S35, S38, and S39, which correspond to locations that are not overlapped with physical objects.

To analyze the effect on subjects responses due to age gap and differences in previous experience with the technology, we divided the subjects into two pairs of different groups, namely groups G1 and G2 and groups P1 and P2. Group G1 and G2 were formed by splitting the participants equally in two groups of eight people. Group G1 consists of subjects in the age range 22–24 while subjects in the age range 25–40 form the group G2. In Table 3, anticipated, omitted, and completed responses mean values are shown. Due to cluttered real environment scene, younger age groups subject have higher mean values as compared to the older age group. Some subjects had already used augmented reality tools. To assess the impact, if any, of prior experience on the number of anticipated, omitted, and correct stimulus responses, we divided the subjects into two groups: P1 and P2, of 10 and 6 people, respectively. More specifically, people in group P1 had tried such systems once or twice while those in group P2 had tried them more than 3 times. In Table 4, anticipated, omitted, and completed mean values are shown. An ANOVA test with 95% confidence interval was applied on the means of the different age groups (G1 and G2) and prior experience groups (P1 and P2), but found no significant difference among these groups. In our collected data we found no difference in age and prior experience on human reaction time in a mixed reality environment. ANOVA *p*-values are shown in Table 5. This could probably be related to the relatively small age difference between the two groups (G1 mean = 22.375, G2 mean = 29.5). Further studies will be needed to assess the role of age in influencing reaction time in MR.

**Table 3:** Average values of responses for different age groups G1 and G2.

**Responses**	**Group**	**Mean**	**Std**
Anticipated	G1	3.12	2.03
	G2	2.5	2.2
Omitted	G1	6.52	3.99
	G2	5	5.10
Completed	G1	30.87	5.96
	G2	32.75	7.11

**Table 4:** Average values of responses from different previous experiences groups P1 and P2.

**Responses**	**Group**	**Mean**	**Std**
Anticipated	P1	3	1.85
	P2	2.62	1.45
Omitted	P1	6.62	1.77
	P2	4.62	1.22
Correct	P1	30.62	2.12
	P2	33	1.09

**Table 5:** ANOVA *p*-values comparison on age and previous experience.

**Comparison of groups**	**Anticipated responses**	**Omitted responses**	**Correct responses**
G1 vs. G2	0.5	0.59	0.57
P1 vs. P2	0.73	0.38	0.47

## 6. Conclusions and future work

This work presented an analysis of the human reaction time in a mixed reality environment, and of the impact of the real scene on its perception. In fact, advanced interaction modalities for interacting and manipulating the mixed reality environment require special motor skills that affect the accuracy of the task and cause longer reaction time. Subjects required a longer time due to the head movement needed for identifying visual stimuli placed outside of the FoV, which is another factor affecting the reaction time and the accuracy of the interaction. Saliency of the real environment is another important factor influencing reaction time in mixed reality: subjects attention toward the real world affects the task accuracy and the total reaction time. For control room application short response times and interaction modalities with high pointing accuracy are needed. This preliminary study suggests that MR can be used in a control room, keeping in mind, however, that the presence of a non-uniform background may have an impact on the operator's reaction time.

## Data availability statement

The raw data supporting the conclusions of this article will be made available by the authors, without undue reservation.

## Ethics statement

Ethical review and approval was not required for the study on human participants in accordance with the local legislation and institutional requirements. The patients/participants provided their written informed consent to participate in this study.

## Author contributions

SA, MC, and FB contributed to conception and design of the study. SA performed the experiments, organized the database, and wrote the first draft of the manuscript. MB performed statistical analysis and revised the first draft. All authors contributed to manuscript revision, read, and approved the submitted version.

## Funding

This project has received funding from the European Union's Horizon 2020 research and innovation programme under the Marie Sklodowska-Curie grant agreement No. 764951.

## Conflict of interest

The authors declare that the research was conducted in the absence of any commercial or financial relationships that could be construed as a potential conflict of interest.

## Publisher's note

All claims expressed in this article are solely those of the authors and do not necessarily represent those of their affiliated organizations, or those of the publisher, the editors and the reviewers. Any product that may be evaluated in this article, or claim that may be made by its manufacturer, is not guaranteed or endorsed by the publisher.
